# Cardiac Reverse Remodelling by 2D and 3D Echocardiography in Heart Failure Patients Treated with Sacubitril/Valsartan

**DOI:** 10.3390/diagnostics11101845

**Published:** 2021-10-06

**Authors:** Valentina Mantegazza, Valentina Volpato, Massimo Mapelli, Valentina Sassi, Elisabetta Salvioni, Irene Mattavelli, Gloria Tamborini, Piergiuseppe Agostoni, Mauro Pepi

**Affiliations:** 1 Centro Cardiologico Monzino IRCCS, Via Parea 4, 20138 Milan, Italy; valevolpato@hotmail.it (V.V.); massimo.mapelli@cardiologicomonzino.it (M.M.); valentina.alba.sassi@gmail.com (V.S.); elisabetta.salvioni@cardiologicomonzino.it (E.S.); irene.mattavelli@cardiologicomonzino.it (I.M.); gloria.tamborini@cardiologicomonzino.it (G.T.); piergiuseppe.agostoni@cardiologicomonzino.it (P.A.); mauro.pepi@cardiologicomonzino.it (M.P.); 2 Department of Clinical Sciences and Community Health, Cardiovascular Section, University of Milan, 20122 Milan, Italy

**Keywords:** sacubitril/valsartan, heart failure with reduced ejection fraction, heart failure aetiology, reverse remodelling, 3D echocardiography

## Abstract

In terms of sacubitril/valsartan (S/V)-induced changes in heart failure with reduced ejection fraction (HFrEF) via three-dimensional (3D) transthoracic echocardiography (TTE) and S/V effects based on HF aetiology, data are lacking. We prospectively enrolled 51 HFrEF patients (24 ischaemic, 27 non-ischaemic). At baseline and at 6-month follow-up (6MFU) after S/V treatment optimisation, we assessed the N-terminal pro-B-type natriuretic peptide (NT-proBNP), and cardiac remodelling by two-dimensional (2D) and 3DTTE. In non-ischaemic patients, 2D and 3DTTE showed an improvement in left ventricular (LV) size and biventricular function at 6MFU vs. baseline: 3D-LV end-diastolic volume (EDV) 103 ± 30 vs. 125 ± 32 mL/m^2^ (*p* < 0.05), 3D-LV ejection fraction (EF) 40 ± 9 vs. 32 ± 5% (*p* < 0.05), right ventricular (RV) 3D-EF 48.4 ± 6.5 vs. 44.3 ± 7.5% (*p* < 0.05); only the 3D method detected RV size reduction: 3D-RVEDV 63 ± 27 vs. 71 ± 30 mL/m^2^ (*p* < 0.05). In ischaemic patients, only 3DTTE showed biventricular size and LV function improvement: 3D-LVEDV 112 ± 29 vs. 121 ± 27 mL/m^2^ (*p* < 0.05), 3D-LVEF 35 ± 6 vs. 32 ± 5% (*p* < 0.05), 3D-RVEDV 57 ± 11 vs. 63 ± 14 mL/m^2^ (*p* < 0.05); RV function did not ameliorate. In both ischaemic and non-ischaemic patients, diastolic function and NT-proBNP significantly improved. In HFrEF patients treated with S/V, 3DTTE helps to ascertain subtle changes in heart chambers’ size and function, which have a major impact on HFrEF prognosis. S/V has significantly different effects on LV function in non-ischaemic vs. ischaemic patients.

## 1. Introduction

Myocardial remodelling has a major pathogenetic role in the progression of heart failure with reduced ejection fraction (HFrEF), and it is strictly linked to neurohormonal upregulation, a recognized hallmark of HF worsening [1,2]. Indeed, it is well known that the inhibition of neurohormonal pathways (e.g., the renin–angiotensin–aldosterone and autonomic sympathetic systems) is the therapeutic cornerstone of HFrEF [1,2].

By inhibiting the renin-angiotensin-aldosterone system [2], sacubitril/valsartan (S/V) is proved to ameliorate left ventricular (LV) volumes, LV systolic [1,3,4,5,6,7] and diastolic function [5,6,8,9], and long-term prognosis in HFrEF [10,11,12,13]. A few studies, conducted on a limited number of cases, demonstrated an improvement in right ventricular (RV) function by two-dimensional (2D) transthoracic echocardiography (TTE) [5,8,9]. However, an extensive assessment of S/V-induced changes in all prognostically meaningful parameters [14] by both 2D and three-dimensional (3D) TTE has never been accomplished in HFrEF patients.

The aim of our study was to comprehensively assess echocardiographic indices of reverse remodelling by 2D and 3D TTE before and 6-months after optimisation of S/V therapy in HFrEF patients, grouped according to HF aetiology (ischaemic heart disease, IHD, vs. non-ischaemic heart disease, non-IHD).

## 2. Materials and Methods

### 2.1. Patient Population and Study Design

**Figure 1:**
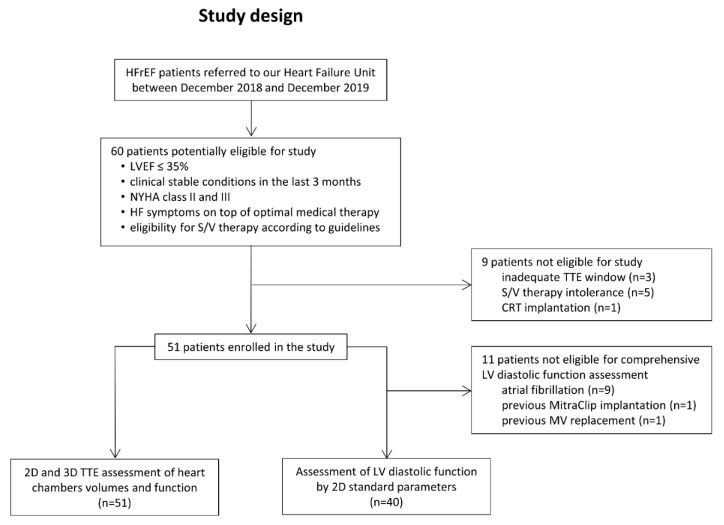
**Study protocol.** CRT = cardiac resynchronization therapy; HF = heart failure; HFrEF = heart failure with reduced ejection fraction; LVEF = left ventricular ejection fraction; MV = mitral valve; S/V = sacubitril/valsartan; TTE = transthoracic echocardiography.

HFrEF patients referred to the Heart Failure Unit of our Institute between December 2018 and December 2019 were evaluated, and were prospectively enrolled if they met the inclusion criteria of the present study: an LV ejection fraction (EF) ≤ 35%; a clinically stable condition for at least 3 months; NYHA class II and III; HF symptoms on top of optimal medical therapy, including angiotensin converting enzyme inhibitors (ACEi) or angiotensin receptor blockers (ARB) at the maximum tolerated dosage; and eligibility for S/V treatment according to guidelines [15]. Exclusion criteria included (i) poor endocardial visualisation; (ii) intolerance or adverse effects to S/V treatment requiring its interruption; and (iii) a necessity of cardiac resynchronization therapy (CRT) during the study observation. To assess the LV diastolic function by 2D parameters, patients were also excluded in the case of arrhythmias during the echocardiographic examination or if they had undergone previous mitral valve repair or replacement (Figure 1).

At baseline, each patient underwent blood tests for N-terminal pro-B type natriuretic peptide (NT-proBNP) dosage, clinical evaluation, and a TTE study the day before interrupting ACEi or ARB. After 36 h, S/V therapy was introduced at a 24/26 mg starting dose, and then progressively up-titrated according to patients’ conditions and consistently with best clinical practice. Repeated efforts were made to increase the S/V dose. Laboratory, clinical, and echocardiographic assessment was repeated at a 6-month follow-up (6MFU) after reaching the maximum tolerated dosage of S/V therapy. Echocardiographic examinations were performed by two operators who were blinded to patients’ clinical data and unaware of the S/V dosage at 6MFU.

The study complied with the Declaration of Helsinki and informed consent was obtained from the enrolled subjects. The present study is part of a multicentre trial on S/V treatment, which was approved in 2018 by the local Ethics Committee (n. R854/18-CCM 898) and registered on ClinicalTrials.gov (NTC04434170).

### 2.2. Echocardiographic Examination

TTE examinations were performed using a Philips ultrasound machine (Epiq CVx-Philips Medical Systems, Andover, MA, USA) equipped with an X5-1 probe. Complete standard 2D TTE analysis was accomplished. Left chambers’ volumes and LVEF were measured by applying the biplane Simpson’s method [16]. RV dimensions and function were calculated using the apical RV-focused view; specifically, RV basal and mid diameters, long axis, indexed end-diastolic area, tricuspid annular plane systolic excursion (TAPSE), and fractional area change (FAC) were measured [16]. Systolic pulmonary artery pressure (sPAP) was derived from the peak velocity of tricuspid regurgitation (TR) and the estimated right atrial pressure, as previously described [17]. The mitral and tricuspid valvular regurgitation grade was assessed by integrating semi-quantitative and quantitative methods [18]. Each patient was assigned a diastolic dysfunction (DD) grade, applying the algorithm for the estimation of LV filling pressures validated in patients with depressed LVEF [19]. This included the mitral valve pulsed wave inflow pattern for the determination of the peak E wave, peak A velocity, the E/A ratio, tissue Doppler-derived medial and lateral mitral annular e’ velocity, average E/e’ ratio, TR velocity, and maximal 2D left atrial (LA) volume index.

**Figure 2:**
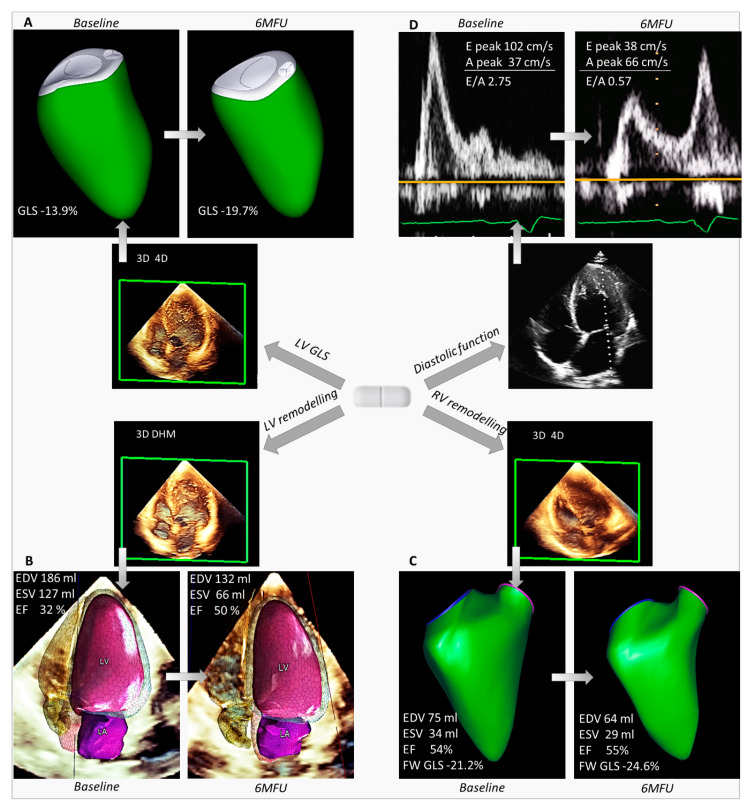
Evaluation of biventricular size and function by 3D transthoracic echocardiography and LV diastolic function by 2D Doppler parameters at baseline and 6-month follow-up after sacubitril/valsartan optimisation. Reported is the example of a patient with non-ischaemic HFrEF, showing significant improvement at 6MFU of (**A**) LV GLS, (**B**) LV volumes and LVEF, (**C**) RV volumes and function, and (**D**) diastolic function by 2D mitral valve inflow pattern. 6MFU = 6-month follow-up; EDV = end-diastolic volume; EF = ejection fraction; ESV = end-systolic volume; FW = free-wall; GLS = global longitudinal strain; LV = left ventricular; RV = right ventricular.

3D image acquisitions included both machine-learning-based and conventional 3D analyses. Single-beat, high-frame-rate, 3D datasets (HM ACQ key on the Epiq system, Philips Medical Systems, Andover, MA, USA) were acquired from the apical 4-chamber view during a single breath-hold [20]. The entire LV and LA cavities were included in the dataset. Offline automated analysis of the left heart chambers was performed using the Dynamic HeartModel software (DHM, Philips Medical Systems, Andover, MA, USA), as previously described [20,21]. For each patient, LV volumes, LVEF, LV mass, and maximal LA volume were recorded. In the case of atrial fibrillation, measurements were averaged over 3 beats. Moreover, conventional 3D acquisitions of the LV from the apical position and of the RV from an apical RV-focused view were performed: the 4-beat full-volume mode was used in patients in sinus rhythm, whereas the single-beat mode was used in atrial fibrillation. Conventional 3D datasets were analysed offline for 3D LV global longitudinal strain estimation (4D LV-Analysis, TomTec Imaging Systems, Unterschleissheim, Germany) and the quantification of 3D RV volumes, RVEF, and RV free-wall (FW) longitudinal strain (4D RV-Function, TomTec Imaging Systems, Unterschleissheim, Germany) [22]. An example of 2D and 3D TTE analysis is presented in Figure 2.

### 2.3. Statistical Analysis

To assess the clinical data and echocardiographic indices of reverse remodelling after optimisation of S/V therapy in HFrEF, grouped according to HF aetiology, the following analyses were applied. Continuous data are presented as mean ± standard deviation or median [interquartile range], after testing for normal distribution with the Kolmogorov–Smirnov test. Categorical variables are expressed as frequencies and percentages. Differences in baseline characteristics, clinical, and echocardiographic parameters between IHD and non-IHD were assessed as follows: continuous variables were compared using the unpaired Student’s *t* test or Mann–Whitney U test, whereas the chi-squared test or Fisher’s exact test were used for categorical variables, as appropriate.

Comparisons between baseline and 6MFU data in the overall population and each aetiologic subgroup were performed by the paired Student’s *t* test or Wilcoxon signed-rank test for continuous variables. Categorical variables were compared with the chi-squared test (or Fisher’s exact if the cell count was <5) or the Friedman test, as appropriate.

All results were considered significant with a *p*-value < 0.05. Statistics were performed with SPSS 27 (SPSS Inc., Chicago, IL, USA).

## 3. Results

### 3.1. Baseline Characteristics of Study Population

Sixty patients who met the inclusion criteria were considered suitable for the present study. Three patients were excluded because of low-quality 3D datasets (inadequate acoustic window), five for symptomatic hypotension after S/V introduction, and one due to CRT implantation during the study period. Thus, our final population included 51 patients (47% IHD and 53% non-IHD). The diastolic function was assessable in a subset of patients (n = 40), since its evaluation was not feasible in nine cases due to atrial fibrillation, in one case for previous MitraClip implantation, and in one patient due to previous prosthetic mitral valve implantation (Figure 1).

Demographic characteristics of the study population are shown in Table 1. At baseline, no differences were noted in comorbidities between the IHD and non-IHD groups, nor was the pharmacological treatment significantly different, with the exception of antiplatelet therapy, which was more frequently prescribed in ischaemic patients.

### 3.2. Clinical and Echocardiographic Data

Clinical and echocardiographic data at baseline and 6MFU in the whole population and in each aetiologic group are reported in Table 2 and Table 3.

**Figure 3:**
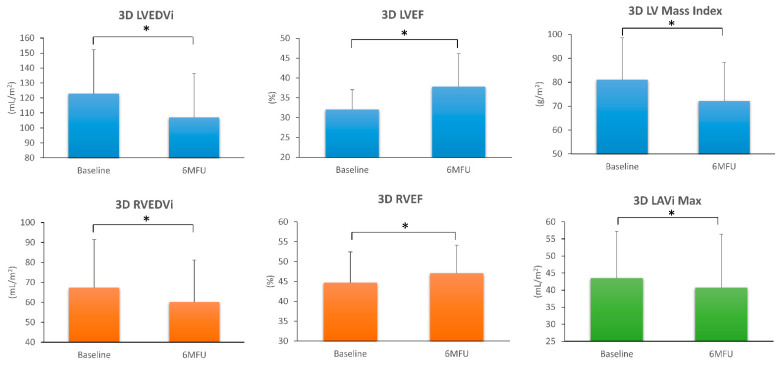
Changes in left ventricular (blue), left atrial (green), and right ventricular (orange) 3D echocardiographic parameters observed in the overall population, 6 months after sacubitril/valsartan therapy. 6MFU = 6-month follow-up; LAVi Max = maximal left atrial volume indexed; LV = left ventricle; LVEDVi = left ventricular end-diastolic volume indexed; LVEF = left ventricular ejection fraction; RVEDVi = right ventricular end-diastolic volume indexed; RVEF = right ventricular ejection fraction. * *p* < 0.05 6MFU vs. Baseline.

2D TTE showed a significant improvement in LV dimensions and function, as well as MR grade, only in non-ischaemic patients. By 3D TTE, we observed improvement in RV dimensions, LV size, and LVEF in both aetiologic groups whereas RV function improved only in non-ischaemic patients (Table 2). Specifically, 3D LVEDV decreased from 124.5 ± 31.6 to 103.0 ± 29.5 mL/m^2^ in non-IHD (*p* < 0.001) and from 120.8 ± 27.3 to 111.7 ± 29.3 mL/m^2^ in IHD (*p* = 0.001). 3D RVEDV decreased from 71.0 ± 29.8 to 62.6 ± 26.6 mL/m^2^ and from 62.8 ± 14.2 to 57.0 ± 11.2 mL/m^2^, respectively, in non-IHD (*p* = 0.003) and IHD (*p* = 0.020). 3D LVEF increased from 31.7 ± 5.1 to 40.3 ± 9.3% in non-IHD (*p* < 0.001), and from 32.4 ± 5 to 34.8 ± 5.6% in IHD (*p* = 0.025). 3D RVEF increased from 44.3 ± 7.5 to 48.4 ± 6.5% in non-IHD (*p* = 0.002). Average changes in LV, LA, and RV 3D parameters in the overall population after 6 months of optimised S/V therapy are shown in Figure 3.

Patients with elevated LA pressure decreased from 40% to 10% in non-IHD and from 45% to 20% in IHD (*p* < 0.05). Finally, S/V induced a significant reduction in NT-proBNP in both ischaemic and non-ischaemic patients (*p* < 0.001 and *p* = 0.009, respectively).

### 3.3. Follow-Up

The mean follow-up after the initiation of S/V lasted 267 ± 43 days (259 ± 39 days in IHD vs. 274 ± 46 days in non-IHD, *p* = NS). In 8 patients, the maximum tolerated S/V dosage was 49/51 mg bd, while the remaining 43 patients reached the target treatment dosage of 97/103 mg bd (19 IHD vs. 24 non-IHD, *p* = NS).

## 4. Discussion

Growing evidence has emerged of the role of S/V in modifying the clinical course of HFrEF patients, through its modulation of neurohormonal imbalance [2]. The effect of S/V on left chambers’ remodelling has been previously demonstrated by traditional 2D TTE [1,3,4,5,6,7,23] and speckle tracking analysis [5]. On the contrary, there is a paucity of data on the impact of S/V on RV dimensions and function, which are based exclusively on 2D echocardiographic parameters [5,9]. The major novelty of our study is a comprehensive evaluation of changes in all prognostically significant echocardiographic parameters [14] after the optimisation of S/V therapy in HFrEF patients, both ischaemic and non-ischaemic, including (i) an assessment of left heart chambers’ and RV size and systolic function both by 2D and 3D echocardiography, (ii) non-invasive estimation of LV filling pressures and sPAP, and (iii) quantification of valve function.

IHD and non-IHD groups were almost equal in size and did not differ significantly at baseline in terms of 2D and 3D echocardiographic parameters, except for a significantly lower TAPSE in IHD compared with non-IHD patients, likely due to the higher number of patients in the IHD group undergoing previous cardiac surgery (mainly coronary artery bypass grafting), which is known to impair the RV longitudinal systolic function [24], or, to a lesser extent, due to previous acute myocardial infarction by right coronary artery occlusion. Notably, the 3D RV FW strain at baseline was significantly higher in IHD vs. non-IHD. Indeed, the longitudinal strain does not necessarily parallel TAPSE, since TAPSE extrapolates the shortening of the entire RV wall from one single point, whereas strain is the expression of the longitudinal deformation of the three segments of the RV FW.

Compared with baseline, 3D LV mass significantly decreased in both IHD and non-IHD, in line with former preclinical [25] and clinical trials [3,4] that reported an S/V-induced reduction in LV mass by 2D TTE in HF patients, suggesting this might be due to the ability of S/V to inhibit abnormal myocardial hypertrophy and fibrosis [4,26]. Additionally, a significant reduction in LV volumes, an increase in 3D LV longitudinal strain, and 2D and 3D LVEF was observed at 6MFU in the overall population and the non-IHD group. A significant reduction in LV dimensions associated with a slight, though significant, LVEF improvement was documented in IHD only by 3D TTE (Table 2). Indeed, in the case of previous myocardial infarction, the LV may undergo profound morphological changes with distortion of its 3D shape, which may be unnoticed by 2D echocardiography [27,28]. Although LV reverse remodelling has been described by 2D TTE at mid- and long-term follow-up after S/V initiation in HFrEF patients [1,3,4,5,6,7,23], only one study conducted subgroup analyses and found no differences in the 2D LVEF improvement between IHD and non-IHD [29]. Such discrepancy from our results may be ascribed to the short follow-up (3 months) and the low dose of S/V achieved by patients enrolled in the study by Almufleh et al. [29]. Due to the significant improvement in LVEF in non-IHD patients (9.5% median increase in 3D LVEF), it could be speculated that device (ICD or CRT) implantation for primary prevention and/or heart transplantation or LV assist device (LVAD) implantation could be prevented or at least delayed in these patients after the introduction of S/V therapy at the maximum tolerated dose, as recently suggested [30].

Concerning the RV, the 2D evaluation did not show significant changes in size, whilst 3D TTE showed a significant reduction in RV volumes in the overall population and in both IHD and non-IHD patients. With S/V, RV systolic function improved significantly at 6MFU vs. baseline in the overall population and non-IHD patients, as expressed by FAC, 3D RVEF, and 3D RV FW strain (Table 2). RV systolic dysfunction has a consistent impact on symptom severity, quality of life, exercise capacity, and survival in HF patients [31,32], and it significantly affects the outcome of patients undergoing LVAD implantation. The assessment of RV dimensions and function by 2D TTE may be inaccurate because of RV crescent-shape morphology. As 3D imaging techniques do not rely on geometric assumptions, they are considered the gold standard for the RV evaluation [28,31,32], and when cardiac magnetic resonance is contraindicated or not feasible, 3D TTE is the mainstay for the RV evaluation [33]. Considering its prognostic role and implications for possible LVAD implantation in HF patients, the RV morphology and function should be routinely evaluated and monitored over time by both 2D and 3D echocardiography.

HFrEF is frequently associated with DD, which has a major impact on morbidity and mortality, irrespective of systolic function [34]. Previous trials showed that S/V has beneficial haemodynamic effects in HF patients with preserved EF [35], though at present, data on the haemodynamic effect of S/V in HFrEF are scarce and conflicting. Through direct pulmonary artery measurements, Khan et al. found that pulmonary artery pressure significantly decreased in 13 outpatients after 1-week treatment with S/V [8]. Other studies used TTE and showed contrasting results regarding changes in Doppler-based diastolic parameters in HFrEF patients on S/V treatment [5,9,29]. The haemodynamic profile of our patients was assessed by integrating all conventional 2D TTE indices of diastolic function [19,34]. A significant improvement in each parameter, as well as in DD class, was shown at 6MFU vs. baseline in the whole population. When the analysis was performed by aetiologic groups, statistically significant amelioration could not be demonstrated in all diastolic indices, probably due to the limited number of cases in each group. Nonetheless, by integrating all these parameters as currently recommended [19], the DD class significantly improved at 6MFU vs. baseline both in IHD and non-IHD (Table 3). Though LVEF improvement in IHD patients might be limited (2.8% median increase in 3D LVEF), these patients experienced a significant haemodynamic improvement, suggesting it is also worth implementing S/V in ischaemic patients still having symptoms in spite of optimal medical therapy.

In line with the aforementioned results, a significant reduction in NTproBNP, a surrogate of biventricular stretching, was detected in both aetiologic groups. We also noticed a decreasing trend in SPAP, although statistical significance did not emerge, probably because SPAP could be accurately calculated in only 39 patients.

Finally, a significant reduction in LA volume, indicative of reverse LA remodelling [3,35], and an improvement in MR grade and NYHA class were observed in the overall population and non-IHD patients, probably because of both improved diastolic function and a more conspicuous LV reverse remodelling in IHD vs. non-IHD cases.

## 5. Conclusions

Beneficial effects on biventricular size and function, as well as on LV filling pressures and NTproBNP levels, were observed in HFrEF patients. However, the impact of S/V on LV and RV systolic function was significantly different between ischaemic and non-ischaemic aetiology (non-ischaemic patients experienced the major improvement in biventricular function). Cardiac reverse remodelling in HFrEF needs to be accurately evaluated to define prognosis and establish a therapeutic approach tailored to the individual patient. In this context, 3D TTE seems to add value to routine 2D TTE, since it allows a better evaluation of RV dimensions and function, as well as the detection of subtle changes in LV size and function undetected by 2D imaging.

### Study Limitations

Some limitations have to be acknowledged. First, the present subanalysis of a multicentre prospective study on S/V was performed only in one HF unit with a non-randomized design, so the small size of the study population might have influenced our results. Second, comparisons with other imaging techniques were not performed. Third, we enrolled stable, ambulatory patients in NYHA class II and III, with a low prevalence of ≥moderate MR, significant pulmonary hypertension, and RV dysfunction, therefore our data cannot be applied to patients with more advanced disease or patients with preserved LVEF. Finally, S/V therapy was titrated to the maximum or intermedium dose in all patients. Therefore, our results may not hold true for HFrEF patients that tolerate S/V at a low dosage. Large multicentre trials enrolling patients in heterogeneous clinical conditions are needed to possibly extend our findings to different HF populations.

## Figures and Tables

**Table 1:** Baseline characteristics of study population.

	Overall Population (*n* = 51)	IHD (*n* = 24)	Non-IHD (*n* = 27)	*p* Value
Age (y)	65 ± 10	66 ± 9	64 ± 10	NS
Male (*n*, %)	43 (84.3)	21 (87.5)	22 (81.5)	NS
BMI (kg/m^2^)	26.2 ± 4.2	26.3 ± 3.7	26.0 ± 4.6	NS
Hypertension (*n*, %)	32 (62.7)	13 (54.2)	19 (70.4)	NS
Dyslipidaemia (*n*, %)	32 (62.7)	18 (75.0)	14 (51.9)	NS
COPD (*n*, %)	2 (3.9)	1 (4.2)	1 (3.7)	NS
Smoking history (*n*, %)	35 (68.6)	21 (87.5)	14 (51.9)	0.008
CKD Stage (*n*, %)				NS
I	14 (27.5)	4 (16.6)	10 (37.0)	
II	16 (31.4)	10 (41.7)	6 (22.2)	
III	21 (41.2)	10 (41.7)	11 (40.7)	
Hyperuricemia (*n*, %)	10 (19.6)	4 (16.6)	6 (22.2)	NS
Diabetes mellitus (*n*, %)	9 (17.6)	5 (20.8)	4 (14.8)	NS
Previous cardiac surgery (*n*, %)	7 (13.7)	6 (25.0)	1 (3.7)	0.042
Previous PCI (*n*, %)	22 (43.1)	21 (87.5)	1 (3.7)	<0.001
Previous MitraClip procedure	1 (2.0)	1 (4.2)	0	NS
History of atrial fibrillation (*n*, %)	14 (27.4)	4 (16.6)	10 (37.0)	NS
LBBB (*n*, %)	14 (27.5)	2 (8.3)	12 (44.4)	0.005
Device therapy (*n*, %)				
ICD	14 (27.5)	11 (45.8)	3 (11.1)	0.011
CRT-P	1 (2.0)	0	1 (3.7)	NS
CRT-D	10 (19.6)	4 (16.6)	6 (22.2)	NS
CCB (*n*, %)	1 (2.0)	0	1 (3.7)	NS
α Blockers (*n*, %)	1 (2.0)	0	1 (3.7)	NS
Ivabradin (*n*, %)	7 (13.7)	4 (16.6)	3 (11.1)	NS
Digoxin (*n*, %)	6 (11.8)	3 (12.5)	3 (11.1)	NS
β Blockers (*n*, %)	51 (100.0)	24 (100.0)	27 (100.0)	NA
ACEi (*n*, %)	36 (70.6)	16 (66.7)	21 (77.8)	NS
ARB (*n*, %)	13 (25.5)	8 (33.3)	6 (22.2)	NS
MRA (*n*, %)	39 (76.5)	17 (70.8)	22 (81.5)	NS
Nitrates (*n*, %)	1 (2.0)	1 (4.2)	0	NS
Loop diuretic (*n*, %)	42 (82.4)	20 (83.3)	22 (81.5)	NS
Thiazide (*n*, %)	2 (3.9)	1 (4.2)	1 (3.7)	NS
Antiplatelet (*n*, %)	36 (70.6)	23 (95.8)	13 (48.1)	<0.001
OAC (*n*, %)	19 (37.3)	12 (50.0)	7 (25.9)	NS

ACEi = angiotensin converting enzyme inhibitor; ARB = angiotensin receptor blocker; BMI = body mass index; CCB = calcium channel blockers; CKD = chronic kidney disease; COPD = chronic obstructive pulmonary disease; CRT-D = cardiac resynchronization therapy-defibrillator; CRT-P = cardiac resynchronization therapy-pacemaker; ICD = implantable cardioverter defibrillator; IHD = ischaemic heart disease; LBBB = left bundle branch block; MRA = mineralocorticoid receptor antagonist; NA = not applicable; Non-IHD = non-ischaemic heart disease; NS = non-significant; OAC = oral anticoagulant therapy; PCI = percutaneous coronary intervention. *p* value is referred to comparisons between IHD and non-IHD patients.

**Table 2:** Clinical and echocardiographic features of the overall population and ischaemic vs. non-ischaemic patients at baseline and 6-month follow-up.

	Baseline			6MFU		
	All (*n* = 51)	IHD (*n* = 24)	Non-IHD (*n* = 27)	All (*n* = 51)	IHD (*n* = 24)	Non-IHD (*n* = 27)
**Clinical Variables**						
NTproBNP (pg/mL)	1225 [661–2896]	1111 [404–2239]	1543 [761–4288]	915 * [370–1812]	869 * [377–1492]	941 * [300–2845]
SBP (mmHg)	119 ± 17	118 ± 21	120 ± 14	107 ± 14 *	106 ± 13 *	107 ± 14 *
HR (bpm)	67 ± 10	63 ± 8	71 ± 10 ^†^	65 ± 10	63 ± 10	67 ± 10
NYHA Class						
II	40 (78.4)	19 (79.2)	21 (77.8)	47 (92.2) *	22 (91.7)	25 (92.6) *
III	11 (21.6)	5 (20.8)	6 (22.2)	4 (7.8) *	2 (8.3)	2 (7.4) *
**2DTTE Variables**						
LAVi (mL/m^2^)	47.7 ± 14.6	46.9 ± 16.1	48.4 ± 13.3	44.4 ± 13.4 *	44.2 ± 14.2	44.5 ± 12.9 *
LV EDVi (mL/m^2^)	101.8 ± 28.6	102.6 ± 29.9	101.0 ± 28.0	92.1 ± 27.6 *	97.9 ± 26.5	87.0 ± 28.0 *
LV ESVi (mL/m^2^)	70.0 ± 23.0	71.1 ± 23.6	69.0 ± 22.9	59.5 ± 25.0 *	66.0 ± 23.0 *	53.8 ± 25.8 *
LV EF (%)	31.9 ± 4.5	31.4 ± 5.1	32.3 ± 3.9	37.1 ± 8.9 *	33.7 ± 7.2	40.1 ± 9.2 *^,†^
MR grade						
<moderate	40 (78.4)	20 (83.3)	20 (74.1)	46 (90.2) *	22 (91.7)	24 (88.9) *
≥moderate	11 (21.6)	4 (16.7)	7 (25.9)	5 (9.8) *	2 (8.3)	3 (11.1) *
TAPSE (mm)	19.8 ± 4.2	18.5 ± 4.5	20.9 ± 3.7 ^†^	19.5 ± 4.6	18.0 ± 3.7	20.8 ± 5.0 ^†^
RVD1 (mm)	42.2 ± 7.8	41.2 ± 5.6	43.0 ± 9.3	40.4 ± 8.3 *	39.8 ± 7.1	41 ± 9.4
RVD2 (mm)	30.9 ± 7.4	29.6 ± 5.6	32.1 ± 8.7	29.6 ± 6.7	28.1 ± 5.9	30.8 ± 7.7
RVD3 (mm)	78.5 ± 11.3	76.5 ± 12.5	80.3 ± 10.2	79.3 ± 9.2	77.5 ± 7.2	81.0 ± 10.6
RV EDAi (cm^2^/m^2^)	12.0 ± 3.8	11.3 ± 2.5	12.7 ± 4.6	11.7 ± 3.4	10.7 ± 2.2	12.5 ± 4.2
RV ESAi (cm^2^/m^2^)	7.4 ± 2.7	7.0 ± 2.5	7.8 ± 3.3	6.7 ± 2.6 *	6.3 ± 1.9 *	7.1 ± 3.0
RV FAC (%)	39.3 ± 9.5	38.9 ± 9.9	39.7 ± 9.3	43.0 ± 9.1 *	42.2 ± 8.7	43.8 ± 9.5 *
TR grade						
<moderate	43 (84.3)	23 (95.8)	20 (74.1)	46 (90.2)	23 (95.8)	23 (85.2)
≥moderate	8 (15.7)	1 (4.2)	7 (25.9)	5 (9.8)	1 (4.2)	4 (14.8)
SPAP (mmHg)	38.1 ± 12.4	39.9 ± 13.3	36.8 ± 11.9	34.6 ± 10.1	34.9 ± 10.3	34.4 ± 10.2
**3DTTE Variables**						
LAVi max (mL/m^2^)	43.9 ± 13.7	42.0 ± 12.4	45.5 ± 14.8	40.7 ± 15.7 *	40.5 ± 14.5	40.8 ± 16.8 *
LV EDVi (mL/m^2^)	122.8 ± 29.4	120.8 ± 27.3	124.5 ± 31.6	107.0 ± 29.4 *	111.7 ± 29.3 *	103.0 ± 29.5 *
LV ESVi (mL/m^2^)	84.3 ± 24.9	82.4 ± 22.7	86.0 ± 27.0	68.2 ± 26.7 *	73.9 ± 25.3 *	63.3 ± 27.3 *
LV EF (%)	32.0 ± 5.0	32.4 ± 5.0	31.7 ± 5.1	37.8 ± 8.3 *	34.8 ± 5.6 *	40.3 ± 9.3 *^,†^
LV GLS (%)	−10.0 ± 2.8	−9.3 ± 2.6	−10.6 ± 2.8	−12.2 ± 4.4 *	−10.1 ± 3.6	−14.3 ± 4.1 *^,†^
LV Mass (g/m^2^)	81.1 ± 17.5	80.2 ± 14.0	81.9 ± 20.3	72.2 ± 16.0 *	72.8 ± 13.0 *	71.6 ± 18.4 *
RV EDVi (mL/m^2^)	67.3 ± 24.2	62.8 ± 14.2	71.0 ± 29.8	60.1 ± 21.1 *	57.0 ± 11.2 *	62.6 ± 26.6 *
RV ESVi (mL/m^2^)	37.4 ± 14.2	34.4 ± 8.8	39.9 ± 17.2	32.1 ± 12.6 *	31.6 ± 9.2 *	32.6 ± 15.0 *
RV EF (%)	44.7 ± 7.8	45.1 ± 8.3	44.3 ± 7.5	47.1 ± 7.1 *	45.4 ± 7.5	48.4 ± 6.5 *
RV FW strain (%)	−22.0 ± 6.3	−24.0 ± 7.3	−20.5 ± 4.8 ^†^	−25.8 ± 7.1 *	−25.6 ± 7.0	−26.0 ± 7.4 *

EDAi = end-diastolic area indexed; EDVi = end-diastolic volume indexed; EF = ejection fraction; ESAi = end-systolic area indexed; ESVi = end-systolic volume indexed; FAC = fractional area change; FW = free wall, GLS = global longitudinal strain; HR = heart rate; IHD = ischaemic heart disease; LAVi = left atrial volume indexed; LAVi max = maximal left atrial volume indexed; LV = left ventricle; MR = mitral regurgitation; Non-IHD = non-ischaemic heart disease; RV = right ventricle; RVD1 = right ventricular basal diameter; RVD2 = right ventricular mid diameter; RVD3 = right ventricular long axis; SBP = systolic blood pressure; sPAP = systolic pulmonary artery pressure; SVi = stroke volume indexed; TAPSE = tricuspid annular plane systolic excursion; TR = tricuspid regurgitation. * *p* < 0.05 6MFU vs. Baseline. ^†^ *p* < 0.05 Non-IHD vs. IHD.

**Table 3:** Diastolic function indices of the overall population and ischaemic vs. non-ischaemic patients at baseline and 6-month follow-up.

	Baseline			6MFU		
	All (*n* = 40)	IHD (*n* = 20)	Non-IHD (*n* = 20)	All (*n* = 40)	IHD (*n* = 20)	Non-IHD (*n* = 20)
E wave	63 [46–79]	64 [52–79]	63 [42–87]	54 [43–62] *	49 [40–64] *	57 [46–62]
A wave	61 [35–82]	66 [29–85]	58 [45–82]	75 [51–92] *	72 [43–85]	79 [59–98] *
E/A	0.8 [0.6–2.4]	0.8 [0.6–2.5]	0.8 [0.6–2.2]	0.6 [0.5–0.9] *	0.7 [0.6–1.1] *	0.6 [0.6–0.7]
E/e’ average	11 [8–15]]	12 [9–16]	9 [6–15]	10 [8–14] *	9 [8–12] *	10 [7–14]
Diastolic dysfunction (n, %)						
Class I	23 (57.5)	11 (55.0)	12 (60.0)	34 (85.0) *	16 (80.0) *	18 (90.0) *
Class II	4 (10.0)	1 (5.0)	3 (15.0)	2 (5.0) *	2 (10.0) *	0 *
Class III	13 (32.5)	8 (40.0)	5 (25.0)	4 (10.0) *	2 (10.0) *	2 (10.0) *

3D = three-dimensional; 6MFU = 6-month follow-up; IHD = ischaemic heart disease; LAVi max = maximal left atrial volume indexed; Non-IHD = non-ischaemic heart disease. * *p* < 0.05 6MFU vs. Baseline.

## Data Availability

Data are available on reasonable request. All data relevant to the study are included in the article.
